# Supplementary material for: Collective language creativity as a trade-off between priming and antipriming
Source: PLoS One. 2021 Nov 29;16(11):e0259285. doi: 10.1371/journal.pone.0259285 (PMC8629240; doi:10.1371/journal.pone.0259285)
Supplement: Attachment — Submitted filename: PONE_rebuttal_letter.pdf [file pone.0259285.s003.pdf]

Dear editors and reviewers,

please accept my sincere gratitude for all your comments and suggestions. They helped to improve the paper a lot! Please find my responses below.

#	**Reviewers' comments**	**Responses**	**Lines in the marked-up manuscript**
1	Definition of the creativity measure. I was unsure as to what was meant by the number of repeated words. For example, would the string "A B A B" have two repeated types (A and B) or four repeated tokens (A twice, B twice). Similarly with pairs: by pair do you mean consecutive words, or all pairs of words? If the former, do they overlap; if the latter does order matter? Again, taking the above string, I could construe the following as possible sets of pairs: {AB,AB} {AB,BA,AB}, {AB,AA,AB,BA,BB,AB},{AB,BA,AA,AB,BA,BA,AB,BB,AB,BA} and possibly other examples. Again, the meaning of repeated also needs to be specified, e.g, in the case {AB,BA,AB} they may be either two or three repeated pairs, depending on whether we are counting types and tokens. I think it would help to provide some short sample texts with the complexity measure calculated, perhaps even examples from the experiments, so the reader is absolutely sure as to how this measure is calculated. I think this is important because the measure features so heavily in the analysis. (Actually, re-reading the manuscript I think this is a between-text rather than a within-text measure, which certainly needs to be clarified)	The respective passage has been rewritten.	217–249

2	Related, I was unsure as to why, in line 212, q=1 if there are no repeated words. My interpretation is that w=p=0 in that case, which would lead to undefined q.	The formula has been corrected and explained.	217–224
3	Similarly, at the end of Study 1, it would help to provide concrete examples of the transformation of comments into vectors to make this more concrete.	An example is provided.	469–472
4	The formulae on lines 445 and 568 have dotted square boxes that show up in various places. I think these should simply not be there, rather than represent missing symbols, but these need to be cleaned up.	Corrected.	498, 623
5	I felt that the conclusions were perhaps limited in scope, mostly recapitulating the specific findings of the study. I think, given the framing in terms of a general conflict between conformity/priming and creativity in the introduction, it would be natural to discuss implications within this context in the conclusion.	The conclusion section has been revised, two paragraphs discussing some limitations of the project have been added. Yet, given a somewhat intricate structure and density of the paper, I feel that summarising the main findings of the study is very important.	675–692
6	Please ensure that your manuscript meets PLOS ONE's style requirements, including those for file naming.	I tried hard to comply with the style guide requirements.	—
7	For this single-authored manuscript, please replace "we" with "I".	Done.	—
8	We note that Figure 1 and 8 includes an image of a participant in the study. If you are unable to obtain consent from the subject of the photograph, please either instruct us to remove the figure or supply a replacement figure by return e-mail for which you hold the relevant copyright permissions and subject consents.	Figure 1 has been removed, Figure 8 has been replaced with a schematic image.	—

9	If you have cited papers that have been retracted, please include the rationale for doing so in the manuscript text, or remove these references and replace them with relevant current references.	I am not aware of any retracted papers on our reference list.	433–439
10	Line 47: There is no reason for creating the acronym CALU for ‘creative aspect of language use’, it is only used twice in the whole manuscript.	Corrected.	47–52
11	Line 73: The author poses the question of why priming and creative language use don’t cancel each other out? I’m not sure what this would look like in practice? After all, people have only two choices, either repeat language forms used previously by their interlocutors or not. What is meant by the processes cancelling each other out?	Corrected.	72–73
12	The paragraph beginning at line 83 should be clarified, perhaps by providing some concrete examples of troll language. From the Sherlock Holmes example, it’s difficult to understand what the author means by an “anomalous distribution of repeated words” characterising the language of internet trolls. The connections aren’t obvious.	The paragraph has been rewritten.	86–105
13	Line 95, first sentence: The author refers to a “theory”, but it isn’t clear what theory is being referenced. It would really help the reader if the theory with regards to “troll-like” comments or reviews were explained more explicitly. It’s also not conceptually clear why a certain number of repetitions should characterise only troll speech. Calling it a “troll effect”, especially if it’s also true to neutral comments that people produce when exposed to other people’s comments seems inaccurate.	Theory is perhaps too strong a word. I tried to elucidate the passage. However, it is just going over what has already been published, so I tried to make it as compact as possible.<br>As for the ‘troll effect’, that is exactly why I put it in quotes and use only as a linking device between what has been observed earlier and what is stated now.	86–106

14	Was there a reason to conduct the study in both English and Russian? If it's only to see if any effect would be true cross-linguistically, this should be made clear. It's not obvious if we should be expecting different effects in the two languages in terms of the initial hypothesis. A lot of effort is made by the author to try to explain the differences in the findings between English and Russian, and minimise them, but I wasn't sure if there was something in the way English and Russian is structured or used in writing that could have contributed to the difference.	Done. As for the second part of the question, an honest answer would be: I don't know. In the paper, it is stated that 'to find out whether the choice of one of those two scenarios depends upon language or some experimental variation that remained unaccounted for, further research is needed.'	153–155
15	Line 135: It's not helpful to co-opt a word that's generally used in a different context in the literature. Using 'antipriming' for the absence of priming is a bit confusing and misleading. The author seems to use it interchangeably with creative language use. Why not just stick with the latter?	Some changes have been made. It would be really a pity to relinquish the term 'antipriming' completely since it helps to see the ambivalent, compromise nature of the language creativity as I understand it.	147–150
16	The samples for analysis in the first study were created by taking an ever increasing range of comments and choosing 50 of them randomly, starting with 50. This should mean that when the range is small, each random selection has a lesser effect on the data than when the range is bigger. Could this be responsible for the larger fluctuation at higher sample indices? Calculating the CCC for a number of randomisations and taking an average might be a better reflection of the true variability of the CCC as the sample index grows.	Thank you for this wonderful idea! I tried it out; the respective figure has been changed and the respective passage rewritten.	282–294

17	Paragraph starting at line 298: It would be very helpful if the author could provide at least one example of how the findings on maximal cliques relate to the data. What does a maximal clique look like in English? If there are fewer maximal cliques in English at the outset, does that mean that words are less likely to be specific to a particular context and more likely to be reused across the comment? I'm trying to figure out what the data show about how the two languages may operate in the comments, but I'm having a hard time.	I tried to clarify the issue, among other things, by including a scheme of the two models of network development.	418–423
18	Reading the comments was completely optional for the participants. Did the author collect any data about whether (or how much) people actually paid attention to previous comments? If so, was there individual variation in this? If participants have to scroll down to the bottom to find their input row, would they pay more attention to the first couple of comments and the last couple of comments? Or just the few closest in time to their own comments? Would the author expect this to have an influence on the outcome? I know this isn't crucial to understanding the findings, but I wonder if it might have some explanatory power. Or if it could be the source of some of the variation?	That is a really great point. I did not collect any data about whether (or how much) people actually paid attention to previous comments, but it definitely could be the source of some variation. A short discussion of this limitation has been included in the conclusions section.	683–689
19	Line 471: A "the" is missing before "situation"	Corrected.	526
20	Line 604: "of" should be "or"	Corrected.	658
21	Sometimes the alone condition is referred to as Alone and sometimes as Condition 1 on the graphs. Similarly, the other condition can be Together or Condition 2. It would be good to standardise this.	Labelling of respective figures has been standardised.	484–485, 587–588

22	I’m not sure Figures 11 & 12 are necessary to include. There is nothing in the left-hand panels and the right hand panels are also not very informative and could probably be described in the text easily. This is optional, but I don’t think they add much to the visualisation of the data, unlike all the other graphs.	The figures have been removed.	512–513
